# Should Diseases Be Named by Their Discoverers?

**Published:** 1918-11-16

**Authors:** 


### Should Diseases be Named by their Discoverers?

### SIR DOUGLAS POWELL AND THE HEINE-MEDIN DISEASE.

Presiding at the second of Dr. Crookshank's Chadwick Lectures on "The Story of a New Disease," Sir Richard Douglas Powell said :—

"In Dr. Crookshank's learned and able exposition of the history and nature of the Heine-Medin disease, which he gave us recently under the title of 'The Story of a New Disease,' he points out that it is the old story of a disease that has long been with us, but has recently attracted fresh attention and found a new name. I confess to a personal objection to diseases bearing the names of their discoverers. It does not seem a graceful way of distinguishing a man to attach a disease to his name: it raises too many questions of priority, which are always undignified and vexatious. Moreover it is a cause of confusion. Dr. Bright did not invent interstitial nephritis, nor, so far as I know, did he suffer from it. Why, then, should it be called Bright's disease, as it in no way belonged to him. Both Heine and Medin very probably suffered many times from influenza, but I am not aware that either of them had that peculiar phase of the disease to which acute polio-myelitis is attributed.

"Dr. Crookshank brought out," the speaker added, "two important points, among others, in his able address. He showed, first, that clinicians too much emphasised one very prominent lesion of the disease acute polio-myelitis—viz., the focal lesion which is responsible for infantile paralysis, whereas other portions of the spinal tract, even including the cerebral areas, are known to be attacked, especially in the epidemic form of the disease; when so attacked, nervous and mental symptoms and paralysis of other limbs, of the facial nerve and of muscles under still more central control are created. The second point, which he illustrated by abundant references to historical records even back to the thirteenth century, is remarkable, the almost constant association of the disease with an attack of influenza, and the correspondence in time of epidemic outbreaks of acute polio-myelitis with epidemics of influenza, a coincidence which leads him to infer that the nervous disease is really one phase or manifestation of influenzal poison."

Sir Douglas Powell trusted that he had not misinterpreted Dr. Crookshank in this summation of the principal ideas gathered from his interesting discourse. The moral would seem to be, Sir Douglas added, scrutinise more carefully cases of influenza—a multifarious disease, and probably a syndrome of many diseases yet to be accurately demarcated.

